# Association between ambient particulate matter concentration and fetal growth restriction stratified by maternal employment

**DOI:** 10.1186/s12884-019-2401-9

**Published:** 2019-07-15

**Authors:** Seung-Ah Choe, Jiyeong Jang, Min Jung Kim, Yoon-Bae Jun, Sun-Young Kim

**Affiliations:** 1 Department of Obstetrics and Gynaecology, CHA University School of Medicine, Gyeonggi-do, 11160 Korea; 2 Department of Epidemiology, Graduate School of Public Health, Brown University, Providence, RI 02903 USA; 3 Division of Epidemiology and Biostatistics, University of Illinois at Chicago, Chicago, IL 60607 USA; 4 Department of Statistics, Seoul National University, Seoul, 08826 South Korea; 5 Department of Cancer Control and Population Health, Graduate School of Cancer Science and Policy, National Cancer Center, Goyang-si, Gyeonggi-do 10408 Korea

**Keywords:** Air pollution, Employment, Particulate matter, Low birth weight, Small for gestational age

## Abstract

**Background:**

Fetal growth has been known to be associated with particulate matter (PM) air pollution during gestation. Given that regular working may deviate outdoor air pollution exposure, the association between air pollution and fetal growth restriction can be different across maternal working status. This study was to assess possible effect modification by maternal employment in the association between exposure to PM during pregnancy and fetal growth restriction.

**Methods:**

Using hourly PM less than or equal to 10 and 2.5 μm in diameter (PM_10_ and PM_2.5_) regulatory monitoring data for 2001–2012 and 2008–2012, respectively, and birth certificate data for 2002–2012, we computed maternal exposures with district-level averages of PM_10_ and PM_2.5_ during one year before birth, entire pregnancy, and the 1st, 2nd and 3rd trimesters. The outcomes of fetal growth restriction were assessed by small for gestational age (SGA, weighted <10th percentile in the same gestational age) as well as low birth weight (LBW, < 2.5 kg) at term. We performed logistic regression to examine the association between PM and each of fetal growth restriction outcomes adjusting for individual risk factors. For effect modification by maternal employment, we estimated adjusted odds ratio (OR) of SGA or LBW for interquartile (IQR) increases in PM_10_ or PM_2.5_ stratified by employed and non-employed mothers. We also computed relative excess risk due to interaction (RERI) to investigate additive interaction.

**Results:**

Among 824,011 singleton term births, 34.0% (279,856) were employed and 66.0% (544,155) were non-employed mothers. Proportions of LBW were 1.5% in employed and 1.6% in non-employed (*P* < 0.001). SGA occurred in 12.7% of employed and 12.8% of non- employed (*P* = 0.124) mothers. For non-employed mothers, we observed increased odds of SGA per IQR increase in PM_10_ for one year before birth (OR = 1.02, 95% confidence intervals (CI): 1.00–1.04, *P* = 0.028). ORs of SGA for full pregnancy period and the 3rd trimester were also positive but did not reach statistical significance. We did not observe positive association for PM_2.5_. RERI was not significant both for PM_10_ and PM_2.5_.

**Conclusions:**

We did not observe evidence of effect modification by maternal employment in the association between ambient PM and fetal growth restriction. Future studies using more refined exposure measures should confirm this finding.

**Electronic supplementary material:**

The online version of this article (10.1186/s12884-019-2401-9) contains supplementary material, which is available to authorized users.

## Background

Fetal growth is assessed by comparing birthweight of the newborn with expected weight for the baby’s gestational age. Specifically, low birth weight (LBW, less than 2,500 g regardless of gestational age) [1] and small for gestational age (SGA, below the 10th percentile for the gestational age based on a birthweight-for-gestational-age measure in reference population) have been used as a proxy for perinatal health. Because LBW does not count for gestational age, the relationship between LBW and SGA differs by gestational term. For term births, given that 10th birthweight for 37 weeks of gestation is higher than 2,500 g, all LBW babies are SGA [2]. Being LBW or SGA is an important predictor of morbidity and mortality of newborns and infants and chronic diseases later in life [3, 4]. Proportion of LBW births including preterm ranges from 7.0% in high-income regions to 16.5% in low- and middle-income countries [1]. Prevalence of SGA births is approximately double the prevalence of LBW births globally [5].

Fetal growth restriction is mostly caused by utero-placental dysfunction leading to inadequate supply of nutrients and oxygen to support normal growth of the fetus [6, 7]. Female baby, firstborn, twins, congenital infection (e.g., malaria, HIV or syphilis), or obstetric complications such as hypertension are associated with retarded fetal growth and development, as well as the duration of pregnancy [8, 9]. Several environmental exposures during antenatal period are reported to be associated with higher risk of LBW. Living at high altitudes and exposure to recreational substance (e.g., alcohol, tobacco, or drug abuse) were also reported to be related with smaller fetal size [10, 11].

Among the physical environment, particulate matter (PM) air pollution is recognized as a risk factor for preterm birth and fetal grown disorders [12]. Previous animal and human studies have revealed that high PM exposure was associated with placental inflammatory reaction [13], abnormal trophoblast invasion [14], and reduced placental angiogenesis [15] which lead possible consequences for the impaired fetal growth. Still the underlying mechanism remains unclear [16, 17].

The estimates of term LBW risk for specific trimesters of pregnancy were not consistent [18–20]. Previous systemic analyses revealed positive association between term LBW and ambient concentration of PM smaller than or equal to 10 and 2.5 μm in diameter (PM_10_ and PM_2.5_) exposed during the entire period of pregnancy which was not evident for the 1st or 2nd trimester [18, 20]. It has been attributed to different strategies for exposure assessment, variability of air pollution, and/or residual confounding [21–23]. One of the potential factors that can result in inconsistent findings of air pollution and fatal grown restriction would be working status of mothers. Physical work demands and job stress in employed mothers were related with higher risk of LBW [24]. Longer time spent in transit among working mothers resulting in higher exposure to traffic-related air pollution and noise which is also related with less optimal fetal growth [25]. On the other hand, the actively employed are likely to have a more favorable health status possibly leading to lower risk of fatal growth restriction than general population at large [26]. In addition, there may be possible misclassification of exposure, given that air pollution exposure has been estimated based on mothers’ residential addresses in most epidemiological studies [27, 28].

Despite a potential influence of maternal employment, few studies investigated the role of mothers’ working status in the association between air pollution and fatal growth restriction at term. Common covariates included in many previous studies for adjustment were mother’s age, education, parity, prenatal care/health care coverage, race/ethnicity and the infant’s sex [20, 29–31]. Several studies indicated effect modification in the association between air pollution and adverse birth outcomes by maternal age, smoking, pre-pregnancy BMI and socioeconomic status (SES) [32–34].This study aimed to explore potential effect modification by employment of mothers in the association between PM air pollution exposed for different stages of pregnancy and fetal growth restriction. To confirm our findings, we used two outcomes, LBW and SGA, indicating more and less severe forms of growth restriction for term babies.

## Method

### Data

We obtained national birth certificate data for 2002–2012 in Seoul, Korea, from Statistics Korea (http://kosis.kr/eng/). Seoul, the capital of South Korea, contains one-fifth of the country’s population (10,442,426 in 2012 within an area of 605 km^2^) [35]. Among 1,045,375 singleton live births in Seoul, we selected 842,710 births (80%) delivered at term (between 37 weeks and 0 day and 41 weeks and 6 days) and with available birth weight information. We excluded birth cases with birth weight less than 0.5 kg and higher than 6.0 kg, because it lacks plausibility, extreme maternal age (> 44 years or < 20 years, 0.3%) and missing for maternal employment status (1.4%). The final study population comprises 824,011 births. When comparing those excluded due to missing for maternal employment with those included, LBW was 1.5 and 1.6% in excluded and included mothers, respectively (P for difference = 0.835). SGA occurred more frequently in excluded mothers (13.5% vs 12.7%, *P* = 0.009). Estimated PM_10_ and PM_2.5_ exposure was generally higher in excluded mothers. For example, PM_10_ concentration for one year before birth was 64.7 μg/m^3^ in excluded mothers compared to 57.9 μg/m^3^ in included mothers.

### Definition of fetal growth restriction

According to the birthweight information on birth certificate data, births with birthweight below the 10th percentile for gestational age in Korean reference population [36] were defined as SGA. Gestational age is based on the physician’s final estimate of gestation using ultrasound taken early in pregnancy and the mother’s date of the last menstrual period. SGA was referred in weeks, rounding off to the nearest completed week [36]. Birthweight < 2.5 kg at birth were classified as LBW following the universal definition [1]. As the 10th percentile of birthweight in those with gestational age of 37 weeks was 2.5 kg, SGA contained all cases of LBW.

### PM data and exposure assessment

We obtained hourly PM_10_ and PM_2.5_ concentrations measured at maximum 40 regulatory air pollution monitoring sites in Seoul during 2001–2012 from the National Institute of Environmental Research as described in previous studies [37, 38]. Briefly, we used PM measurements collected only from 25 urban background sites that are deployed to highly populated areas and to monitor population exposures. As urban roadside sites are located next to busy and large roads for monitoring air pollution from traffic sources of major roadways, these sites would not represent residential exposure (Additional file 1: Figure S1). In contrast, urban background monitoring sites are mostly located at the community-service centers in largely populated residential areas. Thus, we excluded measurements at 12 urban roadside sites and included 25 urban background sites only to better represent the level of air pollution exposure for people who were living in residential areas of the district. In the city of Seoul, with the area of 605 km^2^, at least one urban background site is located in each of the 25 districts (*gu’*s, area 10–47 km^2^). We did not included two additional sites in two *gu’*s because these sites operated for a few years. Because relatively small numbers (6–20) of monitoring sites measured PM_2.5_ before 2007, the analysis for PM_2.5_ was restricted to births between 2008 and 2012.

Using hourly PM measurement data, we computed five exposure metrics corresponding to five antenatal periods at the mothers’ home addresses. Because mothers’ home addresses are available at the *gu* level, the PM concentration measured at a single monitoring site in each *gu* was assigned to all mothers residing in the same *gu*. To compute the five metrics, we calculated the 24-h daily averages for days on which > 75% of hourly measurements (18 h) were available at each site. The application of this inclusion criterion for day resulted in exclusion of 6.9% of days for 11 years and at 25 monitoring sties. Then, we averaged daily concentrations over each of the following five pregnancy periods: one year before birth, whole pregnancy, and the 1st, 2nd and 3rd trimesters. To use exposure estimates consistently for a fixed time period, the 1st, 2nd and 3rd trimesters were defined as 0–13^+ 6^, 14^+ 0^–27^+ 6^, and 28^+ 0^–36^+ 6^ weeks, respectively. To derive representative exposure estimates in each period, we computed one year exposures for mothers using the sites with more than 9 months of daily data and three trimester-exposures for those with at least 75% of the data during each trimester available. All 25 urban background sites met the inclusion criteria for each of the five periods.

### Assessment of confounding and effect modification

We used directed acyclic graphs (DAGs) analysis to examine potential confounders and effect modifiers in the association between air pollution and LBW or SGA (Additional file 1: Figure S2). Association between exposure or fetal growth restriction with maternal employment was explored with regression analysis with adjustment for covariates. To assess the effect modification by mother’s working status during pregnancy, we performed stratified analysis by maternal employment status. Non-employed mothers were defined as the retired, housewives, students, or those without work-related activity. Based on the notion of superiority of additive scale to multiplicative scale in terms of estimating the impact of intervention [39], we evaluated additive interaction between maternal employment and PM exposure in the odds of fetal growth restriction. If the strata-specific effect estimates are not homogenous across strata, the effect modification is considered to be present.

### Statistical analysis

We used chi-square test (for categorical variables) and student t-test (for continuous variables) to assess the difference in individual characteristics as well as fetal growth restriction between employed and non-employed mothers. For the association between PM and fetal growth restriction at term (SGA and LBW), we conducted logistic regression using five exposure metrics after adjusting for individual characteristics separately by employed and non-employed mothers. Individual characteristics included birth date (birth year and month), infant sex, maternal education, maternal age, parity (first childbirth or not), birth season, and gestational age. Non-linear association between birth date and term LBW was adjusted by natural cubic spline with 11 degrees of freedom (df) (1 df per year). Odds ratio (OR) for each birth outcome was estimated per interquartile range (IQR) increase in PM_10_ or PM_2.5_.To allow the comparison of effect estimates across different antenatal periods, the IQR computed for the entire pregnancy was consistently applied to the all analyses across the five pregnancy periods. For effect modification, we compared ORs between employed and non-employed mothers. Then, we further calculated the relative excess risk due to interaction (RERI) to assess additive interaction [40].

Our additional analyses investigated the sensitivity and variation of our primary results. First, we investigated whether the difference in the association between employed and non-employed mothers is enlarged, when exposure assessment is spatially coarse as frequently used in previous studies of air pollution and birth outcomes [41, 42]. For this investigation, we compared our results using *gu*-specific exposures with those of spatially constant exposures, based on daily average PM concentrations over Seoul. Second, we applied mixed models to account for correlation of fatal growth restriction within each of the eight district groups (downtown and areas 1 to 7) and examined the robustness of primary results. These eight district groups were classified based on geographical proximity of 25 districts [43]. The analyses were performed using R (ver. 3.0.3; R Development Core Team, Vienna, Austria).

This study was reviewed and approved by the Institutional Review Board of Seoul National University (IRB No. E1503/002–001).

## Results

In our study population of 824,011 singleton term births, SGA and LBW comprised 11.2% (92,068) and 1.5% (12,764), respectively. The mean PM_10_ concentrations of mothers during one year prior to birth, full pregnancy, the 1st, 2nd, and 3rd trimesters were 57.8 (standard deviation = 9.9), 57.3 (10.8), 58.2 (16.5), 57.2 (16.2), and 56.6 (16.6) μg/m^3^, respectively. PM_2.5_ concentrations for births in 2008–2012 were 29.1 (6.7), 28.9 (7.0), 29.3 (8.7), 28.8 (8.5), and 28.4 (8.5) μg/m^3^. PM_10_ concentrations for 1 year prior to birth were moderately correlated with those during the three trimesters (0.44 to 0.62), whereas the correlations of PM_10_ concentrations between trimesters were weak (− 0.09 to 0.26) (Additional file 1: Table S2). In the simple regression of PM or fatal growth restriction on maternal employment, Maternal employment was associated with exposure (regression coefficients: -0.02, *P* < 0.001 for entire pregnancy PM_10_ and − 0.02, *P* < 0.001 for entire pregnancy PM_2.5_) and outcome (− 0.06, *P* < 0.001 for SGA and − 0.10, *P* < 0.001 for LBW) with adjustment for covariates.

**Fig. 1:**
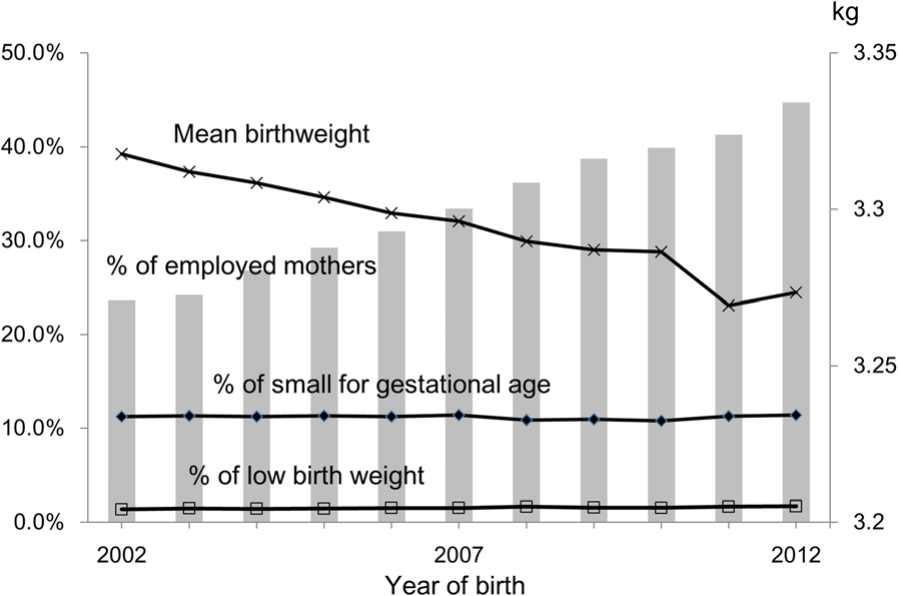
Trends of % of numbers of employed mothers, small for gestational age, and low birth weight and mean birthweight from 2002 through 2012 in 824,011 singleton term births, Seoul, Korea

During the study period from 2002 through 2012, mean birth weight decreased consistently (*β* = − 0.005, *P* < 0.001). The proportion of employed mothers increased from 23.6% in 2002 to 44.7% in 2012. Whereas percentage of LBW increased from 1.4% in 2002 to 1.7% in 2012 (P for trend < 0.001 for both), percentage of SGA was consistent over time (11.2 to 11.4%, P for trend = 0.880) (Fig. 1).

At the time of birth, 34.0% of mothers (279,856) were employed and 66.0% (544,155) were non-employed (Table 1). Proportions of SGA and LBW between employed and non-employed mothers were 12.7 and 12.8% and 1.5 and 1.6%, respectively. Although there was only 1% difference between the two groups, these differences were statistically significant due to the large size of population. SGA and LBW were more likely to occur in female babies in both employed and non-employed mothers. Employed mothers with SGA and LBW tended to have no previous pregnancy experience compared to non-employed mothers with SGA and LBW. Average residential concentrations for PM_10_ and PM_2.5_ were slightly higher in non-employed mothers than in employed mothers for all five gestational periods (Additional file 1: Figure S3).

**Table 1:** Individual characteristics and PM air pollution concentrations of 824,011 singleton term births across three birth status by mothers’ employment status, Seoul, Korea, 2002–2012

	Employed mothers (*n* = 279,856)			Non-employed mothers (*n* = 544,155)		
	Normal	SGA excluding LBW	LBW	Normal	SGA excluding LBW	LBW
	(*n* = 244,414; 87.3%)	(*n* = 31,294; 11.2%)	(*n* = 4,148; 1.5%)	(*n* = 474,765, 87.2%)	(*n* = 60,774, 11.2%)	(*n* = 8,616; 1.6%)
Birthweight (kg)	3.37 ± 0.33	2.77 ± 0.13	2.31 ± 0.16	3.38 ± 0.34	2.76 ± 0.13	2.31 ± 0.17
Female sex, n (%)	114,624 (46.9)	19,313 (61.7)	2,461 (59.3)	211,911 (46.7)	37,086 (61.0)	5,186 (60.2)
Gestational age (weeks)	39.22 ± 1.07	39.29 ± 0.97	38.21 ± 1.06	39.19 ± 1.08	39.28 ± 0.97	38.27 ± 1.09
Maternal age (years)	31.01 ± 3.30	30.84 ± 3.27	31.18 ± 3.50	30.66 ± 3.86	30.39 ± 3.93	30.85 ± 4.20
20–24	3,666 (1.5)	442 (1.4)	69 (1.7)	24,821 (5.2)	3,882 (6.4)	568 (6.6)
25–29	79,446 (32.5)	10,855 (34.7)	1,325 (31.9)	159,036 (33.5)	21,417 (35.2)	2,667 (31)
30–34	126,570 (51.8)	15,911 (50.8)	2,090 (50.3)	217,912 (45.9)	26,808 (44.1)	3,786 (43.9)
35–39	31,667 (13.0)	3,720 (11.9)	574 (13.8)	65,075 (13.7)	7,671 (12.6)	1,377 (16)
≥ 40	3,065 (1.3)	366 (1.2)	90 (2.2)	7,921 (1.7)	996 (1.6)	218 (2.5)
Nulliparity	161,663 (66.1)	23,621 (75.5)	3,143 (75.8)	241,127 (50.8)	37,269 (61.3)	5,363 (62.2)
Maternal education level (years)						
< 7	154 (0.1)	17 (0.1)	5 (0.1)	1,463 (0.3)	257 (0.4)	52 (0.6)
7–12	34,570 (14.1)	4,318 (13.8)	697 (16.8)	180,995 (38.1)	23,271 (38.3)	3,635 (42.2)
> 12	209,690 (85.8)	26,959 (86.1)	3446 (83.1)	292,307 (61.6)	37,246 (61.3)	4,929 (57.2)
Birth season						
Spring	59,540 (24.4)	7,672 (24.5)	981 (23.6)	118,082 (24.9)	15,292 (25.2)	2,176 (25.3)
Summer	58,753 (24.0)	7,363(23.5)	992 (23.9)	112,865 (23.8)	14,306 (23.5)	2,111 (24.5)
Fall	64,437 (26.4)	8,408 (26.9)	1,147 (27.7)	120,076 (25.3)	15,843 (26.1)	2,230 (25.9)
Winter	61,684 (25.2)	7,851 (25.1)	1,028 (24.8)	123,742 (26.1)	15,333 (25.2)	2,099 (24.4)
PM_10_ during pregnancy [μg/m^3^]						
1 year before birth	56.24 ± 9.42	56.22 ± 9.45	55.90 ± 9.24	58.75 ± 10.08	58.8 ± 10.17	58.28 ± 10.20
Entire pregnancy	55.68 ± 10.21	55.65 ± 10.24	55.32 ± 10.16	58.14 ± 10.84	58.20 ± 10.91	57.74 ± 11.00
First trimester	56.75 ± 15.63	56.83 ± 15.68	56.52 ± 15.30	59.06 ± 16.73	59.13 ± 16.87	58.42 ± 16.49
Second trimester	55.52 ± 15.47	55.53 ± 15.57	55.10 ± 15.19	57.85 ± 16.42	58.00 ± 16.50	57.69 ± 16.18
Third trimester	54.85 ± 17.24	54.69 ± 17.30	54.15 ± 17.27	57.58 ± 18.09	57.52 ± 18.17	57.10 ± 18.03
PM_2.5_ during pregnancy [μg/m^3^] ^a^						
1 year before birth	28.35 ± 6.21	28.30 ± 6.25	28.17 ± 5.96	29.60 ± 7.00	29.65 ± 7.08	29.44 ± 7.03
Entire pregnancy	28.10 ± 6.55	28.03 ± 6.59	27.94 ± 6.29	29.32 ± 7.33	29.33 ± 7.39	29.22 ± 7.40
First trimester	28.60 ± 8.12	28.59 ± 8.18	28.40 ± 7.77	29.78 ± 9.04	29.82 ± 9.14	29.61 ± 9.03
Second trimester	28.08 ± 7.95	28.04 ± 7.98	28.84 ± 7.68	29.23 ± 8.81	29.29 ± 8.86	29.22 ± 8.82
Third trimester	27.70 ± 8.51	27.58 ± 8.51	27.39 ± 8.48	28.97 ± 9.31	28.94 ± 9.38	28.83 ± 9.35

The first, second, and third trimesters were defined as 0–13^+ 6^, 14^+ 0^–27^+ 6^, and 28^+ 0^–36^+ 6^ weeks. ^a^Analysis for PM_2.5_ is restricted to births occurred in 2008–2012

ORs of SGA and LBW were generally higher in non-employed mothers than employed mothers (Table 2). ORs of SGA and LBW were close to null in employed mothers for all exposure periods. For non-employed mothers, 13.7 μg/m^3^ (IQR in the full pregnancy period) increase in PM_10_ for one year before birth was associated with 2% increase in odds of SGA (OR = 1.02, 95% confidence intervals (CI): 1.00–1.04, *P* = 0.028). The positive association of PM_10_ for the full pregnancy period and the 3rd trimester with SGA did not reach statistical significance. RERIs indicating additive interaction by maternal employment did not show statistical significance. ORs of LBW were also higher in non-employed mothers than employed mothers, but RERIs were not significant.

**Table 2:** Odds ratios (ORs) and 95% confidence intervals (CIs) for small-for-gestational age (SGA) and low birth weight (LBW) at term per interquartile-increase in PM_10_, stratified by employment status in 824,011 singleton term births in Seoul, Korea for 2002–2012

	District-mean			Seoul-mean		
	Employed	Non-employed	RERI (95% CI)	Employed	Non-employed	RERI (95% CI)
SGA						
One year before birth	1.01 (0.98, 1.04)	1.02 (1.00, 1.04)	-0.02 (− 0.06, 0.01)	0.96 (0.88, 1.05)	1.00 (0.95, 1.06)	− 0.03 (− 0.06, 0.00)
Entire pregnancy	1.01 (0.98, 1.04)	1.02 (1.00, 1.03)	−0.03 (− 0.06, 0.01)	1.02 (0.97, 1.07)	0.98 (0.95, 1.01)	− 0.03 (− 0.06, 0.01)
First trimester	1.01 (0.99, 1.04)	1.00 (0.99, 1.02)	−0.01 (− 0.04, 0.02)	1.02 (0.99, 1.04)	1.00 (0.98, 1.01)	0.01 (− 0.02, 0.04)
Second trimester	1.01 (0.99, 1.03)	1.00 (0.99, 1.02)	−0.01 (− 0.04, 0.02)	1.01 (0.98, 1.03)	0.99 (0.97, 1.00)	−0.03 (− 0.06, 0.00)
Third trimester	1.00 (0.98, 1.03)	1.01 (1.00, 1.03)	−0.01 (− 0.04, 0.02)	0.99 (0.97, 1.02)	1.00 (0.98, 1.01)	−0.03 (− 0.06, 0.00)
LBW						
One year before birth	1.02 (0.94, 1.11)	1.04 (0.99, 1.10)	−0.03 (− 0.12, 0.06)	1.07 (0.83, 1.38)	0.93 (0.81, 1.08)	−0.06 (− 0.15, 0.04)
Entire pregnancy	1.01 (0.93, 1.10)	1.03 (0.98, 1.08)	−0.03 (− 0.12, 0.06)	1.01 (0.88, 1.15)	0.92 (0.85, 1.00)	−0.01 (− 0.10, 0.08)
First trimester	1.00 (0.94, 1.07)	1.01 (0.97, 1.05)	0.00 (−0.08, 0.08)	0.99 (0.92, 1.07)	0.99 (0.94, 1.04)	0.04 (−0.04, 0.11)
Second trimester	0.98 (0.92, 1.05)	1.01 (0.97, 1.05)	0.03 (−0.05, 0.11)	0.97 (0.90, 1.05)	0.95 (0.91, 0.99)	0.01 (−0.07, 0.09)
Third trimester	1.03 (0.96, 1.10)	1.03 (0.99, 1.07)	−0.02 (− 0.10, 0.06)	1.03 (0.95, 1.11)	1.00 (0.96, 1.05)	−0.01 (− 0.09, 0.07)

*RERI* Relative excess risk due to interaction. ORs are adjusted for birth date (birth year and month), infant sex, maternal education, maternal age, parity (first childbirth or not), birth season, and gestational age. All ORs and 95% CIs across five antenatal periods were estimated for an interquartile range of PM_10_ during full year of pregnancy (13.7 μg/m^3^)

The positive association was not found when we used coarse exposure assessment applying citywide Seoul-mean concentration. ORs were lower both for SGA and LBW than those in our primary analysis using district-means and became negative for some exposure periods. Our findings were similar, when we applied mixed models to account for additional variability at the district group level (Additional file 1: Table S2 and S3). Increased odds for SGA per 13.7 μg/m^3^ increase of PM_10_ during one year before birth in non-employed mothers remained significant.

For PM_2.5_, we generally found consistent patterns to those for PM_10_. However, most effect estimates gave null association for both employed and non-employed mothers (Table 3).

**Table 3:** Odds ratios (ORs) and 95% confidence intervals (CIs) for small-for-gestational age (SGA) and low birth weight (LBW) at term per interquartile-increase in PM_2.5_, stratified by employment status, in 386,483 singleton term births in Seoul, Korea for 2008–2012

	District-mean			Seoul-mean		
	Employed	Non-employed	RERI (95% CI)	Employed	Non-employed	RERI (95% CI)
SGA						
One year before birth	0.98 (0.96, 1.01)	1.01 (0.99, 1.02)	−0.02 (−0.06, 0.01)	0.97 (0.90, 1.05)	0.99 (0.94, 1.04)	−0.03 (− 0.06, 0.00)
Entire pregnancy	0.98 (0.96, 1.00)	1.00 (0.98, 1.01)	−0.03 (− 0.07, 0.00)	1.00 (0.94, 1.05)	0.96 (0.93, 1.00)	−0.03 (− 0.06, 0.00)
First trimester	1.00 (0.98, 1.01)	1.00 (0.99, 1.01)	0.00 (−0.04, 0.03)	1.01 (0.99, 1.04)	0.99 (0.98, 1.00)	0.00 (−0.03, 0.03)
Second trimester	0.99 (0.97, 1.01)	1.00 (0.99, 1.01)	−0.02 (− 0.05, 0.02)	1.00 (0.97, 1.03)	0.98 (0.97, 1.00)	−0.03 (− 0.06, 0.00)
Third trimester	0.99 (0.97, 1.01)	1.00 (0.99, 1.01)	0.00 (−0.04, 0.03)	0.99 (0.97, 1.01)	1.00 (0.99, 1.02)	−0.01 (− 0.04, 0.03)
LBW						
One year before birth	0.98 (0.96, 1.01)	1.05 (0.95, 1.17)	−0.05 (− 0.15, 0.05)	0.97 (0.9, 1.05)	0.89 (0.39, 2.04)	−0.07 (− 0.15, 0.02)
Entire pregnancy	0.98 (0.96, 1.00)	1.07 (0.97, 1.18)	−0.09 (− 0.19, 0.01)	1.00 (0.94, 1.05)	1.38 (0.89, 2.13)	−0.07 (− 0.16, 0.01)
First trimester	1.00 (0.98, 1.01)	1.03 (0.96, 1.11)	−0.05 (− 0.15, 0.04)	1.01 (0.99, 1.04)	1.01 (0.88, 1.17)	−0.01 (− 0.09, 0.07)
Second trimester	0.99 (0.97, 1.01)	1.01 (0.94, 1.09)	−0.03 (− 0.12, 0.07)	1.00 (0.97, 1.03)	1.00 (0.87, 1.15)	−0.05 (− 0.13, 0.03)
Third trimester	0.99 (0.97, 1.01)	1.06 (0.98, 1.14)	−0.03 (− 0.13, 0.06)	0.99 (0.97, 1.01)	1.11 (0.95, 1.3)	−0.03 (− 0.13, 0.06)

*RERI* Relative excess risk due to interaction. ORs are adjusted for birth date (birth year and month), infant sex, maternal education, maternal age, parity (first childbirth or not), birth season, and gestational age. All ORs and 95% Cis across five antenatal periods were estimated for an interquartile range of PM_2.5_ during full year of pregnancy (7.8 μg/m^3^) to all estimates

## Discussion

We observed positive associations between PM_10_ concentrations during one year before birth and SGA at term only in non-employed mothers. This association was not evident in those employed. Although we reached different conclusions between employed and non-employed mothers, there was lack of evidence indicating additive interaction by maternal employment status in the association between air pollution and fetal growth restriction. This pattern was consistent but weak for LBW which is stricter definition of fetal growth restriction for term birth. This finding implies that the association between air pollution and fetal growth restriction can be close to null if we use more severe of fetal growth restriction. Based on the homogeneity in the effect estimates across maternal employment strata which is also one of the evidences of confounding [44], our finding indicates a possible role of maternal employment as a confounder in the association between air pollution and fetal growth restriction.

Although there is lack of evidence for maternal employment as an effect modifier, we generally found higher ORs in non-employed mothers than employed mothers. Average estimated concentrations of PM of their residential addresses were slightly higher in mothers without employment compared to those employed consistently across all five antenatal periods. As people living closer to major roads or pollutant-producing facilities were more likely to be at lower SES [45, 46], higher exposure estimates of PM in non-employed mothers may confirm generally lower SES for non-employed mothers than for employed mothers. However, this tendency of exposure to higher air pollution in more socioeconomically deprived group is not universal. In largely populated and congested cities such as Seoul, people may prefer living close to major roads that allow easy access to transportation. A recent study in Seoul showed that children with higher SES were found to be living closer to major roads [43]. As a proxy of social and individual characteristics, maternal employment or working during pregnancy would mean better or worse socioeconomic condition. The adverse health effect of air pollution could be stronger in the socioeconomically deprived people than their counterpart, possibly due to high air pollution exposure, nutritional deficiency, and/or limited access to health care [45]. Several studies found stronger association between air pollution and adverse birth outcomes for mothers living in deprived neighborhoods compared to those in affluent neighborhoods [47, 48], though this finding was not replicated in another study [49]. Considering potential hazard of working such as more work demands, job stress, and higher exposure to traffic-related air pollution, the average effect of lower PM exposure might have been blunted. Future studies should elucidate the pathways between air pollution, fetal growth restriction, and employment focusing on each possible factor.

Among all term singleton births of this study, the proportion of LBW was relatively low. Previous studies reported wide ranges of LBW rate across countries or regions within a country. Prevalence of LBW at term birth was as low as 3.4% in U.S. [50] and 3.0% in U.K. [51], whereas high prevalence was seen as 8.2% in Nepal and 10% in Ethiopia [52]. Across the different regions of U.K., the prevalence ranged widely from 2.6 to 4.0% [51]. In Japan, LBW was 2.7% in 1979 which increased to 5.3% in 2010 [53]. As we restricted our population to those living in Seoul, the capital city with best access to health care, the relatively lower rate of LBW in our study would result from easy healthcare access compared to mothers living in other areas [54]. This considerably low prevalence of LBW, representing a more severe outcome, in our population could have made it difficult to find an association of PM compared to studies that reported associations in other populations. In contrast, SGA, comprising around 10% of the population and regarded as less strict definition of LBW for term births in our study, yielded positive association in non-employed mothers consistent with those in previous studies. This finding suggests that SGA would be a practical measure for fetal birth restriction in the populations with extremely low LBW rate.

The magnitude of the relationship between PM_10_ or PM_2.5_ and LBW at term was lower in our study than in previous studies which employed population-based birth record data [55–57]. In addition to lower LBW, this might be due to potential misclassification driven by the restricted address information in our data. [58]. We used district-averages of air pollution as individual exposures of mothers, given the limited data availability to district-level addresses. This limitation may have affected underestimation of risk estimates, although the urban background monitoring sites mostly located at the community-service centers in largely populated residential areas may well represent the exposure level of residents of the corresponding districts. A study of SGA and LBW using a birth cohort in Vancouver, Canada, showed that fine spatial-scale individual exposures based on full address information and individual exposure assessment approach yielded stronger associations than those based on crude exposures [59], Our finding of lower risk estimates using city-wide average concentrations than those using district-means also suggests the possibility of increasing risks when it is replaced by spatially refined individual exposures. Future studies using extended address information will allow us to assess the association with high validity.

We found negative associations between the 2nd trimester increase in PM_10_ and LBW estimated by Seoul-mean concentrations in non-employed mothers. In general, ORs for the 1st and 2nd trimesters were also slightly lower than those for one year before and entire pregnancy. Considering the result of district-based analysis, this may be incidental finding due to multiple comparison and/or residual confounding effect by spatial variation of exposure by districts. This counter-intuitive association for Seoul-mean may support the value of district-mean as more precise exposure estimate compared to citywide mean.

There are other several limitations in this study. First, there is possible information bias because we did not verify mothers’ self-reported working status during pregnancy. Some of the mothers classified as employed might have taken maternity leave during pregnancy and some of the unemployed might have left their job just before birth. As a retrospective study of birth registry data, we could not assess the duration of employment during pregnancy for each mother. Future studies based on prospectively collected data could minimize this possible misclassification bias. Second, because we used the administrative data, some key individual confounders related to smoking intensity, previous pregnancy history, and type of LBW (constitutional or pathological, and severe or mild) were not available. As there were previous findings of associations between exposure to PM air pollution and smoking intensity, smoking might have confounded air pollution-related health effect [60]. Considering that the smoking rate of women in South Korea is only 5–8% [61], smoking rate in pregnant women would likely be lower and thus smoking would have a negligible impact on our results. Late-onset fetal growth restriction probably represents a more heterogeneous group with less characteristic histological changes [7]**.** If parts of LBW births are early-onset, exploring the association with 2nd or 3rd trimester PM would not be appropriate. As we included only term births, our LBW cases are presumed to be largely late-onset. Considering the potential heterogeneity in our LBW at term cases, future studies should subdivide the births into several groups by potential causes or severity and look at the association. Third, there can be misclassification bias in estimating maternal exposure based on residential address at the time of birth. According to previous report of a population-based sample cohort in Korea, proportion of relocation across district (*gu*) or province was 8–25% during the same period of our study period (2003–2012) [62]. Relocation rate for less than one year could be much smaller particularly in pregnant women. Fourth, maternal PM exposure based on district might have contributed to the general null finding. However, since temporal variability is much larger than spatial variability in daily averages of air pollution exposures in our data [63], the impact of exposure measurement error resulting from spatial misclassification may not be substantial. More refined individual exposure measure would improve the precision of effect estimates. Lastly, generalizability of the findings would be limited because mothers in our study are mostly residents in Seoul which less represent deprived socio-economic conditions which also contribute to poor fetal growth [64].

## Conclusions

Exposure to high ambient PM air pollution during one year before birth tended to increase the risk of SGA at term in non-employed mothers living in Seoul, Korea. Despite potential difference due to mothers’ health status, socioeconomic condition, and exposure misclassification related to their employment status, there was no evidence of effect modification by maternal employment in the association between PM concentration and fetal growth restriction. Futures studies using refined metrics would be helpful to confirm this finding.

## Additional file

Additional file 1: **Table S1.** Correlation coefficients between a pair of the five metrics of mothers’ PM_10_ concentrations corresponding to the five antenatal periods of 824,011 singleton term births in Seoul, Korea, for 2002–2012. **Table S2.** Odds ratios (OR) and 95% confidence intervals (CI) for small-for-gestational age (SGA) and low birth weight (LBW) at term per interquartile-increase in PM_10_^*^ from mixed models that account for correlation of fatal growth restriction at the district group, by employment status in 824,011 singleton term births in Seoul, Korea for 2002–2012. **Table S3.** Odds ratios (ORs) and 95% confidence intervals (CIs) for small-for-gestational age (SGA) and low birth weight (LBW) at term per interquartile-increase in PM_2.5_^*^ from mixed models that account for correlation of fatal growth restriction at the district group, by employment status in 386,483 singleton term births in Seoul, Korea for 2008–2012. **Figure S1.** Map of urban background and urban roadside sites of the regulatory air quality monitoring network in Seoul. **Figure S2.** Directed acyclic graph of individual characteristics that potentially confound the association between residential PM concentration (“exposure”) and fetal growth restriction (“outcome”). **Figure S3.** Average concentrations of PM_10_ and PM_2.5_ between employed and non-employed mothers by five different antenatal periods of singleton term births in Seoul, Korea. (A) PM_10_ (B) PM_2.5. (DOCX 659 kb)_

## Data Availability

The Korean birth data and regulatory air pollution monitoring data that support the findings of this study are available in the Statistics Korea website (https://mdis.kostat.go.kr) and the open data portal website (https://www.airkorea.or.kr/web/last_amb_hour_data?pMENU_NO=123), respectively.

## Abbreviations

LBW: Low birth weight
OR: Odds ratios
PM_10_: Particulate matter ≤10 μm in diameter
PM_2.5_: Particulate matter ≤2.5 μm in diameter
SGA: Small for gestational age
