# Supplementary material for: Association between ambient particulate matter concentration and fetal growth restriction stratified by maternal employment
Source: BMC Pregnancy Childbirth. 2019 Jul 15;19:246. doi: 10.1186/s12884-019-2401-9 (PMC6632189; doi:10.1186/s12884-019-2401-9)
Supplement: Supplementary file 1 — Table S1. Correlation coefficients between a pair of the five metrics of mothers’ PM10 concentrations corresponding to the five antenatal periods of 824,011 singleton term births in Seoul, Korea, for 2002–2012. Table S2. Odds ratios (OR) and 95% confidence intervals (CI) for small-for-gestational age (SGA) and low birth weight (LBW) at term per interquartile-increase in PM10* from mixed models that account for correlation of fatal growth restriction at the district group, by employment status in 824,011 singleton term births in Seoul, Korea for 2002–2012. Table S3. Odds ratios (ORs) and 95% confidence intervals (CIs) for small-for-gestational age (SGA) and low birth weight (LBW) at term per interquartile-increase in PM2.5* from mixed models that account for correlation of fatal growth restriction at the district group, by employment status in 386,483 singleton term births in Seoul, Korea for 2008–2012. Figure S1. Map of urban background and urban roadside sites of the regulatory air quality monitoring network in Seoul. Figure S2. Directed acyclic graph of individual characteristics that potentially confound the association between residential PM concentration (“exposure”) and fetal growth restriction (“outcome”). Figure S3. Average concentrations of PM10 and PM2.5 between employed and non-employed mothers by five different antenatal periods of singleton term births in Seoul, Korea. (A) PM10 (B) PM2.5. (DOCX 659 kb) [file 12884_2019_2401_MOESM1_ESM.docx]

Additional file 1

Table S1. Correlation coefficients between a pair of the five metrics of mothers’ PM_10_ concentrations corresponding to the five antenatal periods of 824,011 singleton term births in Seoul, Korea, for 2002-2012.

| Antenatal period | (1) | (2) | (3) | (4) | (5) |
| --- | --- | --- | --- | --- | --- |
| (1) One year before birth  (2) Entire pregnancy  (3) First trimester  (4) Second trimester  (5) Third trimester | 1  0.90  0.57  0.58  0.58 | 1  0.58  0.78  0.62 | 1  0.26  -0.11 | 1  0.36 | 1 |

Table S2. Odds ratios (OR) and 95% confidence intervals (CI) for small-for-gestational age (SGA) and low birth weight (LBW) at term per interquartile-increase in PM_10_^*^ from mixed models that account for correlation of fatal growth restriction at the district group, by employment status in 824,011 singleton term births in Seoul, Korea for 2002-2012

|  | **Employed mothers** | | | | | | | |  | **Non-employed mothers** | | | | | | |
| --- | --- | --- | --- | --- | --- | --- | --- | --- | --- | --- | --- | --- | --- | --- | --- | --- |
|  | District-mean | | | |  | Seoul-mean | | |  | District-mean | | |  | Seoul-mean | | |
| **SGA** | | OR | 95% CI | |  | OR | 95% CI | |  | OR | 95% CI | |  | OR | 95% CI | |
| One year before birth | | 1.01 | 0.98 | 1.04 |  | 0.96 | 0.88 | 1.04 |  | 1.02 | 1.00 | 1.03 |  | 1.00 | 0.96 | 1.05 |
| Entire pregnancy | | 1.01 | 0.99 | 1.04 |  | 1.02 | 0.97 | 1.07 |  | 1.01 | 0.99 | 1.03 |  | 0.98 | 0.95 | 1.01 |
| First trimester | | 1.01 | 0.99 | 1.04 |  | 1.02 | 0.99 | 1.04 |  | 1.00 | 0.99 | 1.02 |  | 1.00 | 0.98 | 1.01 |
| Second trimester | | 1.01 | 0.99 | 1.03 |  | 1.01 | 0.98 | 1.03 |  | 1.00 | 0.98 | 1.01 |  | **0.99** | **0.97** | **1.00** |
| Third trimester | | 1.00 | 0.98 | 1.03 |  | 0.99 | 0.97 | 1.02 |  | 1.01 | 0.99 | 1.02 |  | 1.00 | 0.98 | 1.01 |
|  | |  |  |  |  |  |  |  |  |  |  |  |  |  |  |  |
| **LBW** | | OR | 95% CI | |  | OR | 95% CI | |  | OR | 95% CI | |  | OR | 95% CI | |
| One year before birth | | 1.02 | 0.94 | 1.11 |  | 1.07 | 0.86 | 1.33 |  | 1.04 | 0.99 | 1.09 |  | 0.94 | 0.84 | 1.05 |
| Entire pregnancy | | 1.01 | 0.93 | 1.10 |  | 1.01 | 0.88 | 1.15 |  | 1.03 | 0.98 | 1.08 |  | **0.92** | **0.85** | **0.99** |
| First trimester | | 1.00 | 0.94 | 1.07 |  | 0.99 | 0.92 | 1.07 |  | 1.01 | 0.97 | 1.05 |  | 0.99 | 0.94 | 1.04 |
| Second trimester | | 0.98 | 0.92 | 1.05 |  | 0.97 | 0.90 | 1.05 |  | 1.00 | 0.96 | 1.05 |  | **0.95** | **0.90** | **1.00** |
| Third trimester | | 1.03 | 0.96 | 1.10 |  | 1.03 | 0.95 | 1.11 |  | 1.03 | 0.98 | 1.07 |  | 1.00 | 0.96 | 1.05 |

RERI, relative excess risk due to interaction. ORs are adjusted for birth date (birth year and month), infant sex, maternal education, maternal age, parity (first childbirth or not), birth season, and gestational age. We applied an interquartile range of PM_10_ during full year of pregnancy (13.7 µg/m^3^) to all estimates. The 8 district groups were defined based on geographical proximity of 25 districts [41];

Table S3. Odds ratios (ORs) and 95% confidence intervals (CIs) for small-for-gestational age (SGA) and low birth weight (LBW) at term per interquartile-increase in PM_2.5_^*^ from mixed models that account for correlation of fatal growth restriction at the district group, by employment status in 386,483 singleton term births in Seoul, Korea for 2008-2012

|  | **Employed mothers** | | | | | | |  | **Non-employed mothers** | | | | | | |
| --- | --- | --- | --- | --- | --- | --- | --- | --- | --- | --- | --- | --- | --- | --- | --- |
|  | District-mean | | |  | Seoul-mean | | |  | District-mean | | |  | Seoul-mean | | |
| **SGA** | OR | 95% CI | |  | OR | 95% CI | |  | OR | 95% CI | |  | OR | 95% CI | |
| One year before birth | 0.99 | 0.96 | 1.01 |  | 1.02 | 0.87 | 1.18 |  | 1.01 | 0.98 | 1.06 |  | 1.03 | 0.91 | 1.17 |
| Entire pregnancy | 0.98 | 0.93 | 1.03 |  | 1.01 | 0.89 | 1.14 |  | 1.01 | 0.97 | 1.05 |  | 0.98 | 0.89 | 1.09 |
| First trimester | 0.99 | 0.96 | 1.03 |  | 1.03 | 0.97 | 1.09 |  | 1.00 | 0.98 | 1.03 |  | 0.99 | 0.95 | 1.04 |
| Second trimester | 1.00 | 0.96 | 1.03 |  | 1.03 | 0.97 | 1.09 |  | 1.00 | 0.97 | 1.02 |  | 0.98 | 0.94 | 1.03 |
| Third trimester | 0.98 | 0.95 | 1.01 |  | 0.96 | 0.92 | 1.01 |  | 1.02 | 0.99 | 1.04 |  | 1.02 | 0.98 | 1.06 |
|  |  |  |  |  |  |  |  |  |  |  |  |  |  |  |  |
| **LBW** | OR | 95% CI | |  | OR | 95% CI | |  | OR |  | |  | OR | 95% CI | |
| One year before birth | 1.03 | 0.90 | 1.17 |  | 0.96 | 0.92 | 1.01 |  | 1.03 | 0.91 | 1.17 |  | 1.02 | 0.98 | 1.06 |
| Entire pregnancy | 1.00 | 0.88 | 1.13 |  | 1.19 | 0.86 | 1.65 |  | 0.98 | 0.89 | 1.09 |  | 0.97 | 0.75 | 1.25 |
| First trimester | 0.98 | 0.89 | 1.08 |  | 1.01 | 0.87 | 1.18 |  | 1.00 | 0.98 | 1.03 |  | 1.01 | 0.89 | 1.14 |
| Second trimester | 0.97 | 0.88 | 1.06 |  | 1.01 | 0.87 | 1.17 |  | 1.00 | 0.97 | 1.02 |  | 0.98 | 0.87 | 1.10 |
| Third trimester | 0.97 | 0.90 | 1.06 |  | 1.03 | 0.91 | 1.16 |  | 1.02 | 0.99 | 1.04 |  | 1.03 | 0.93 | 1.13 |

We applied an interquartile range of PM_2.5_ during full year of pregnancy (7.8 µg/m^3^) to all estimates. The 8 district groups were defined based on geographical proximity of 25 districts [41]


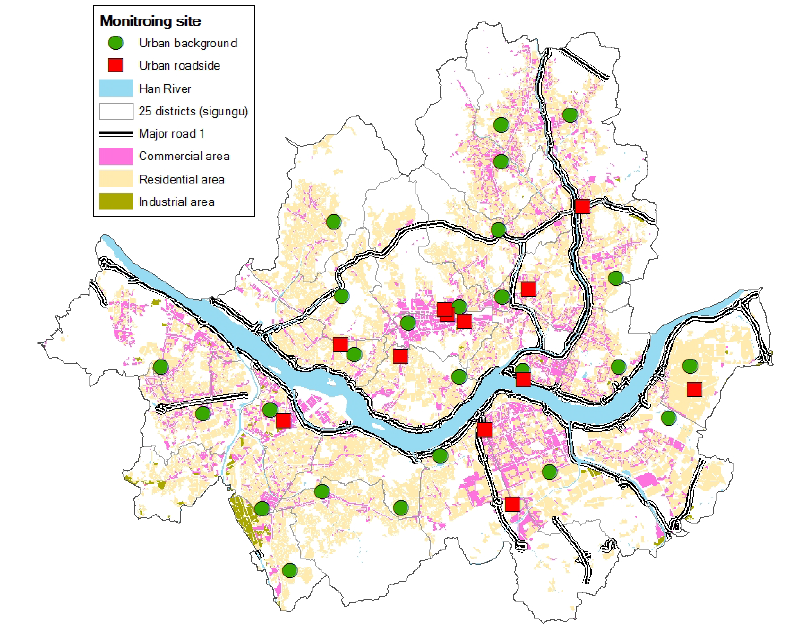


Figure S1. Map of urban background and urban roadside sites of the regulatory air quality monitoring network in Seoul


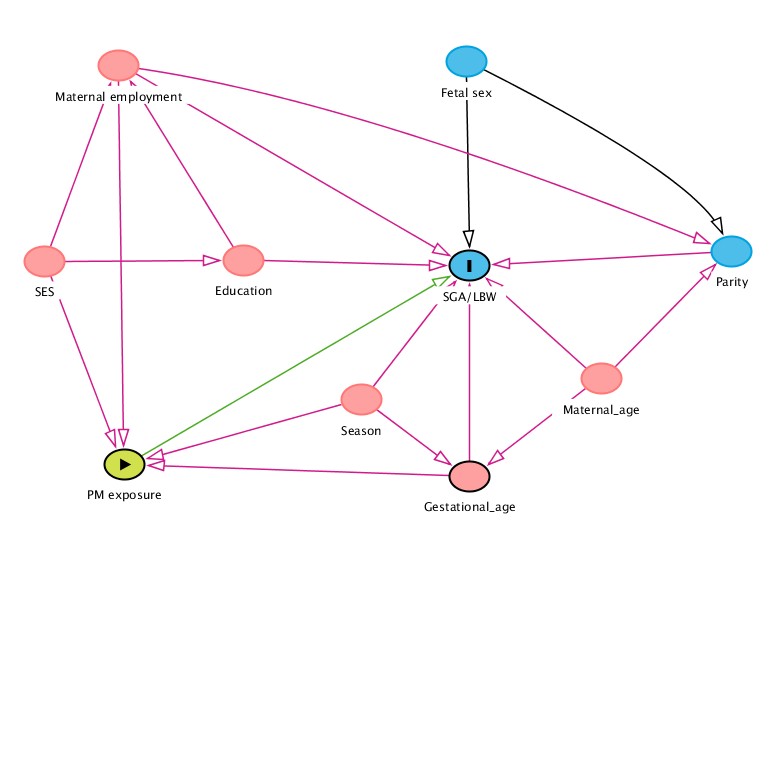


Figure S2. Directed acyclic graph of individual characteristics that potentially confound the association between residential PM concentration (“exposure”) and fetal growth restriction (“outcome”)


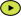
 exposure


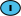
 outcome


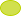
 ancestor of exposure


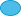
 ancestor of outcome


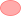
 ancestor of exposure and outcome

SES, socioeconomic status; SGA, small for gestational age; LBW, low birth weight.

(A)
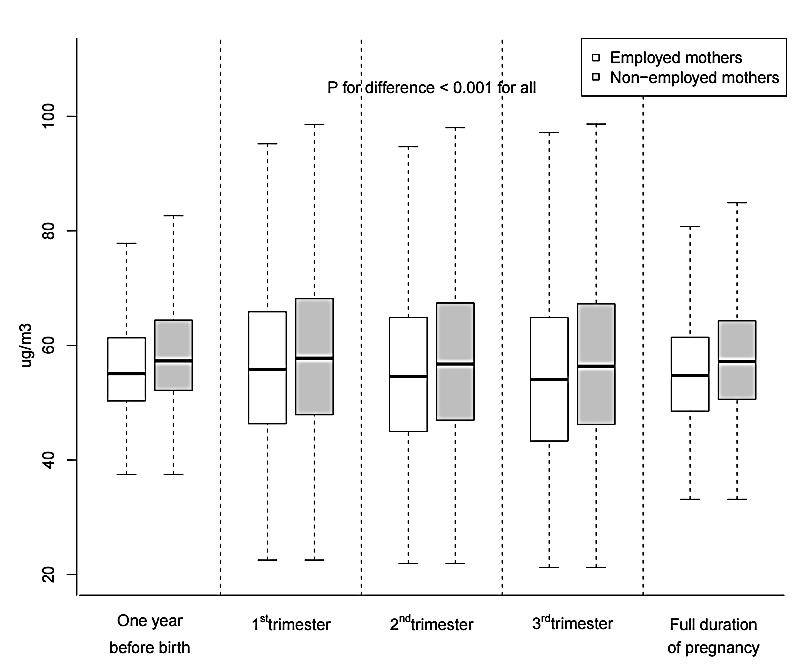
 (B)
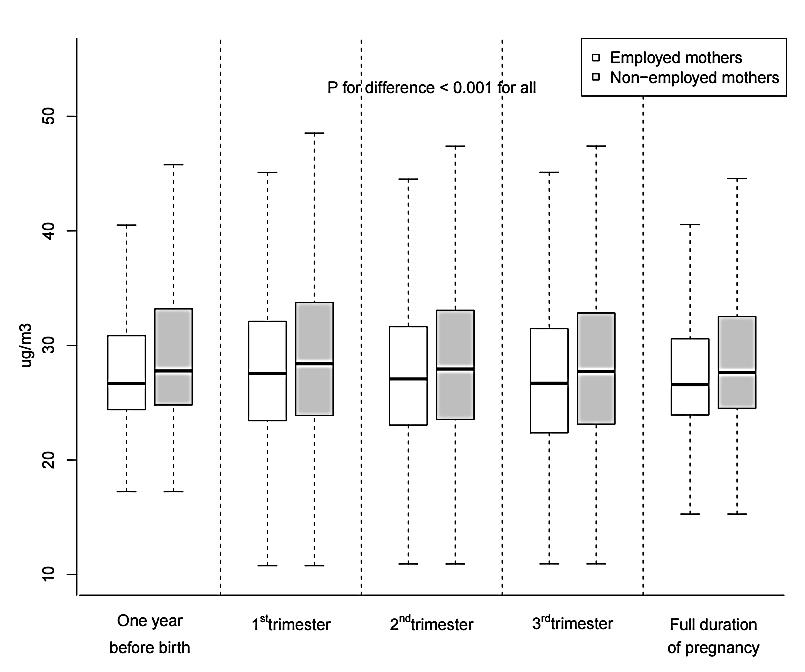


Figure S3. Average concentrations of PM_10_ and PM_2.5_ between employed and non-employed mothers by five different antenatal periods of singleton term births in Seoul, Korea. (A) PM_10_ (B) PM_2.5_
